# Optical Coherence Tomography Imaging: Novel Insights into the Vascular Response After Coronary Stent Implantation

**DOI:** 10.1007/s12410-012-9138-4

**Published:** 2012-05-05

**Authors:** Milosz Jaguszewski, Ulf Landmesser

**Affiliations:** Cardiology, Cardiovascular Center, University Hospital of Zurich, Raemistrassse 100, 8091 Zurich, Switzerland

**Keywords:** Optical coherence tomography, Optical frequency domain imaging, Stent, Coronary intervention, Stent thrombosis, Intravascular imaging

## Abstract

Optical coherence tomography (OCT) is a high-resolution imaging technique that is increasingly used for intracoronary imaging to characterize coronary atherosclerotic plaques and vascular responses after coronary stent implantation. Introduction of optical frequency-domain imaging (OFDI; second generation OCT) has simplified practical use of this novel imaging modality resulting in a more widespread availability in interventional cardiology. Here we highlight recent insights into the acute and chronic vascular response after coronary stent implantation by OCT imaging. OCT provides cross-sectional images with approximately 10-fold higher resolution as compared to intravascular-ultrasound (IVUS), allowing for precise evaluation of tissue coverage and malapposition of coronary stent struts. More than 30 studies using OCT to compare vascular responses to different stents have now been reported. Recent studies have examined the relation between OCT-image characteristics and tissue composition around stent struts. OCT is used for evaluation of novel stent concepts, such as bioengineered stents and bioabsorbable stents, where it provides more accurate information than IVUS. While intracoronary OCT imaging is further developed, including faster 3D-OCT-image-reconstruction, larger OCT studies/registries with standardized analysis will provide more insights into clinical implications of observations from OCT-imaging after coronary stent implantation.

## Introduction

Optical coherence tomography (OCT) is a near-infrared light-based imaging modality [1] that is now quite widely available in interventional cardiology. OCT provides high-resolution cross-sectional images of the inner vascular wall and surface of blood vessels with an approximately 10-fold higher image resolution as compared to intravascular ultrasound (IVUS) [1]. OCT imaging uses an interferometry technique based on time-delay measurements of the light reflected or backscattered from the tissues [1]. There are two processing modes used for intracoronary OCT imaging, i.e. the first generation time-domain OCT imaging systems and the more recently available second generation frequency-domain OCT imaging systems, also called optical frequency domain imaging (OFDI) [2, 3]. OFDI has made the application of OCT in the clinical setting substantially easier, in particular the faster image acquisition as compared to the first-generation time-domain OCT imaging systems [4, 5]. Time-domain OCT and more recently OFDI have been used to analyze the vascular response to coronary stenting in a substantial number of studies, and the present article will summarize important insights from these investigations.

## OCT and Evaluation of Coronary Stent Healing: Focus on Stent Strut Coverage and Strut Malapposition

While first generation drug-eluting stents reduced the need for repeated revascularization, coronary stent healing was also delayed, requiring prolonged double anti-platelet therapy in these patients [6, 7]. Impaired coronary stent healing, in particular the lack of stent endothelialization, has been suggested as one important mechanism causing very late stent thrombosis in patients receiving a first-generation drug-eluting stent (Cypher and Taxus) as suggested by pathological studies [8•]. Several autopsy studies have described a markedly impaired neointimal healing of stent struts in patients who had a fatal late or very late coronary stent thrombosis [9, 10]. Delayed healing was observed in patients after sirolimus and paclitaxel-eluting first-generation stent implantation as compared to BMS implantation as evident by persistent fibrin deposition and substantially impaired stent endothelialization [9, 10].

**Fig. 1:**
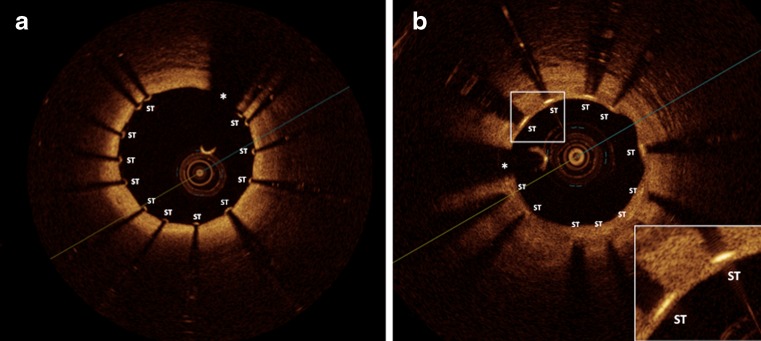
The high resolution of OCT (axial resolution 10–15 μm) allows for detection of thin layers of stent strut coverage and sensitive detection of stent strut apposition/malapposition: **a**, OCT cross-sectional image demonstrating well apposed stent struts; **b**, OCT image demonstrating stent struts covered with a thin neointima layer that is below the IVUS axial resolution (100 μm). *Asterisk* indicates OCT catheter. ST, stent strut

Intravascular ultrasound examination could not detect a thin neointimal coverage in the majority of sirolimus-eluting stent struts in the chronic phase raising the question to what extent these stents indeed remained uncovered [11]. OCT imaging clearly provided a substantially more detailed evaluation of coronary stent strut coverage as compared to the IVUS analysis [11] (Fig. 1.). In an early study of 34 patients who underwent coronary time-domain OCT and IVUS evaluation 6-month after sirolimus-eluting stent implantation the prevalence of covered stent struts as detected by OCT but undetectable by IVUS was 64 % [11]. Therefore, there has been a great interest in the use of OCT for the assessment of the vascular response after coronary stent implantation.

Moreover, intravascular ultrasound (IVUS) studies have previously suggested a high prevalence of incomplete stent apposition (ISA) in patients with very late stent thrombosis after DES implantation [12, 13]. A meta-analysis of IVUS studies who had a baseline exam and a follow-up exam has indicated that the risk of late acquired stent malapposition is substantially increased after DES implantation as compared to BMS implantation, and late stent malapposition was associated with late and very late stent thrombosis [12, 13].

**Fig. 2:**
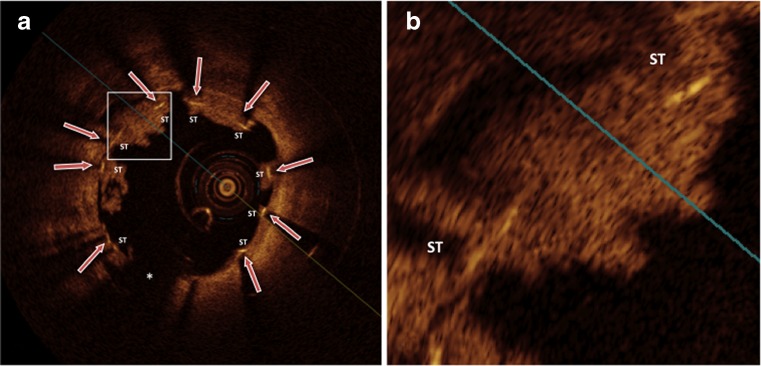
Very late in-stent thrombosis after DES implantation. **a**, malapposed stent struts, that are at least partially uncovered (*red arrows*); **b**, magnification of malapposed stent struts at least partially uncovered or covered by protruding white thrombi. *Asterisk* indicates OCT catheter. ST, stent strut

Due to the high resolution for the identification of coronary stent struts, OCT is likely capable to determine the apposition and degree of malapposition of stents struts in more detail (Fig. 2.). In a study using both, IVUS and OCT, OCT was superior in detection of malapposition of stent struts, likely due to its ability to detect small gaps between stent struts and the vessel wall that may be missed by IVUS [14]. The stent strut is considered malapposed by OCT when the abluminal strut surface is separated from the luminal contour [15••].

Importantly, OCT provides valuable insights providing a potential explanation for the relation between stent malapposition and stent thrombosis. In patients who had undergone first-generation sirolimus-eluting stent implantation it was observed that incomplete stent strut apposition (ISA) was associated with a substantially higher rate of impaired stent healing and with the presence of OCT-detected small thrombi at follow-up, further suggesting that stent strut malapposition and subsequently impaired stent healing represents a substrate for late or very late stent thrombosis [14]. Moreover, another study using OCT in 178 patients at 9 to 13 months follow-up after implantation of different drug-eluting stents supported the notion that a delayed stent strut coverage was substantially more frequently observed (approximately 9-fold) in struts with incomplete stent strut apposition or in non-apposed side-branch stent struts [16]. Gutiérrez-Chico et al. have performed a study examining the relation between acute stent strut malapposition as detected and quantified by OCT and stent strut coverage in the follow-up exams at 6 to 13 months (including analysis of 66 stents) [17]. It was observed that the larger the initial stent strut malapposition the greater the likelihood of a delayed healing in the follow-up [17]. Interestingly, stent struts with small degrees of acute malapposition (< 270 μm) were covered and apposed in the follow-up exam [17].

As described in detail below, several studies have now compared stent strut coverage and strut malapposition for several types of coronary stents (i.e. DES and BMS), different DES at different time points after implantation, and for different clinical indications (i.e. stable CAD vs ACS) by using coronary OCT imaging. However, it needs also to be taken into account, as described in more detail below, that besides the characteristics and design of the stent, the stent implantation technique and the underlying clinical scenario requiring stent implantation (i.e. ACS with thrombotic material vs stable coronary disease) likely also determine the degree and time-line of stent healing.

## Comparison of Stent Strut Coverage and Malapposition Between DES vs BMS, Different DES, or in Patients with Stable CAD vs an Acute Coronary Syndrome

There have now been more than 30 observational or randomized studies reported using OCT to examine stent healing after coronary stent implantation, largely focusing on detection of the percentage of uncovered stent struts and stent struts with malapposition (for summary of randomized studies see Table 1), and some of these recent observations are described below.

**Table 1:** Randomized trials using OCT for evaluation of stent strut coverage and malapposition

Study	Stent type	N	Follow-up (months)	Uncovered stent struts (%)	Stent strut malapposition (%)
DES vs BMS					
Guagliumi et al. [18]	PES vs BMS	118	13	5.7 vs 1.1	0.9 vs 0.1
Guagliumi et al. [19]	SES vs PES vs ZES vs BMS	77	6	8.1 vs 4.1 vs 0.1 vs 0.9	2.3 vs 2.3 vs 0.0 vs 0.1
Guagliumi et al. [20]	ZES vs BMS	44	6	0.0 vs 2.0	0.0 vs 0.15
DES vs DES					
Miyoshi et al. [52]	SES vs PES	27	6	12.7 vs 6.6	1.4 vs 0.5
Moore et al. [53]	Polymer-c. SES vs Non-pol. SES	24	3	11.7 vs 2.8	2.2 vs 1.2
Guagliumi et al. [54]	PES vs B-PES HD	60	6	5.3 vs 7.0	1.4 vs 0.8
Barlis et al. [22]	BES vs SES	56	9	1.8 vs 6.3	
Gutierrez-Chico [23]	BES vs SES	21	24	1.5 vs 1.8	0.1 vs 0.4
Gutierrez-Chico [24]	R-ZES vs EES	58	13	7.4 vs 5.8	1.8 vs 1.4
Takano et al. [25]	EES vs PES	42	6	2.3 vs 5.2	2.1 vs 5.7
DEB					
Gutierrez-Chico [55]	DCB + BMS vs BMS + DCB	26	6	8.1 vs 5.3	

*BES* biolimus-eluting stent, *BMS* bare-metal stent, *B-PES* biolimus-eluting stent with biodegradable polymer, *DCB* drug-coated balloon, *EES* everolimus-eluting stent, *PES* paclitaxel-eluting stent, *PF-SES* polymer-free sirolimus-eluting stent, *R-ZES* zotarolimus-eluting stent with slow-release and Biolynx polymer (Resolute), *SES* sirolimus-eluting stent, *ZES* zotarolimus-eluting stent (Endeavor)

### OCT Studies Comparing DES vs BMS

The largest randomized study to date using OCT to compare the vascular response after implantation of a DES (TAXUS Express, paclitaxel-eluting stent) and an otherwise identical bare-metal stent (Express BMS) is the OCT substudy of the prospective, randomized Harmonizing Outcomes with Revascularization and Stents in Acute Myocardial Infarction (HORIZONS-AMI) trial in patients with ST-segment elevation myocardial infarction (STEMI) that has included 118 consecutive randomized patients [18]. The TAXUS Express, paclitaxel-eluting stents (PESs) reduced neointimal hyperplasia, but had also a higher rate of uncovered and malapposed stent struts as compared with the otherwise identical BMS when examined 13 months after stent implantation [18]. This phenomenon is observed for most drug-eluting stents, that suppress excessive neointima formation (i.e. restenosis), but also have a delayed stent strut healing. The fast-release zotarolimus-eluting stent (Endeavor) tested in the studies by Guagliumi et al. as shown in the DES vs BMS section of Table 1 [19, 20] had also a higher restenosis rate. In fact, the same authors have compared this stent with the later generation of zotarolimus-eluting, slow-release stents (Resolute), that more efficiently suppressed restenosis, and have observed a stronger suppression of the neointimal response but also a higher proportion of uncovered and malapposed stent struts at the 6-month OCT follow-up with the newer generation of the zotarolimus-eluting stents (Resolute) [21].

### OCT Studies Comparing Different DES

Within the LEADERS trial an OCT substudy was performed in 56 patients to compare stent strut coverage following implantation of a biodegradable versus a durable polymer-coated drug-eluting stent [22]. Stent strut coverage at a mean follow-up of 9 months appeared to be more complete in patients after implantation of biodegradable polymer-coated, biolimus-eluting stents (BESs) when compared with the durable polymer-coated sirolimus-eluting stents (SESs) [22]. A long-term follow-up OCT exam suggested that after 24 months the stent strut coverage was similar between the two stent types, i.e. 1.5 % versus 1.8 % uncovered stent struts [23].

An OCT substudy of the RESOLUTE All Comers trial has compared stent healing of the hydrophilic polymer-coated zotarolimus-eluting stent (Resolute) with fluoropolymer-coated everolimus-eluting stent (Xience V) at 13-month follow-up [24]. There were no significant differences observed with respect to stent strut tissue coverage and malapposition at 13-month follow-up, e.g. 7.4 % and 5.8 % uncovered stent struts [24]. A recent prospective, randomized OCT study has compared coronary stent healing after everolimus-eluting stent (EES) and paclitaxel-eluting stent (PES) implantation and has suggested a favorable stent healing response after EES implantation in a 6 months follow-up examination (Table 1) [25]. Several studies using OCT to compare stent healing of different DES are summarized in Table 1, suggesting that besides the stent implantation technique and the clinical scenario the design of DES likely is important for the vascular healing response.

### OCT Examination After DES-Implantation in Stable CAD and Acute MI

**Fig. 3:**
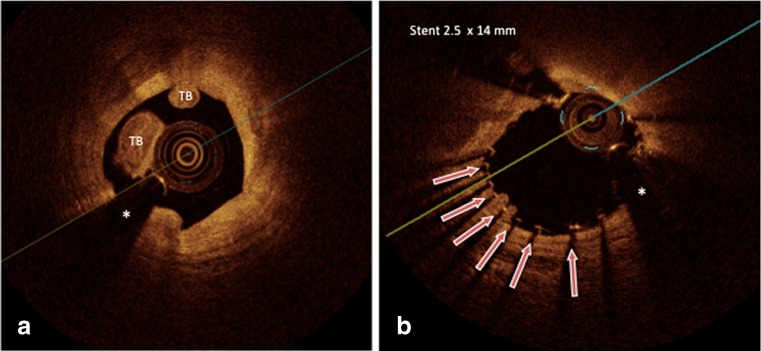
This image illustrates potential aspects of why an acute coronary syndrome may predispose to an impaired stent healing: **a**, Red thrombus in the culprit lesion of the proximal segment of RCX in a patient with NSTEMI; **b**, After stent implantation stent struts with underlying and protruding thrombi are to be seen likely promoting malapposition (*red arrows*). *Asterisk* indicates OCT catheter

Retrospective pathological and clinical coronary OCT imaging studies have observed a higher rate of uncovered and malapposed stent struts in DES that were implanted during an acute STEMI as compared to DES implanted in patients with stable coronary disease [26–28], suggesting that the clinical situation where stent implantation is performed needs to be considered for the interpretation of the stent healing response. Figure 3 shows an OCT in a patient with NSTEMI before and after coronary stent implantation, where thrombotic material is to be seen under stent struts revealing one potential mechanism promoting malapposition in patients with an acute coronary syndrome (Fig. 3).

### OCT Evaluation of New Stent Technologies, eg Bioengineered Stents

OCT is being used to examine whether novel stent technologies may have an accelerated or favorable vascular healing response. The above described data suggest that drug-eluting stents that efficiently suppress restenosis have mostly some degree of delayed stent healing. Therefore there has been substantial interest in designing bioengineered stents with a potential to accelerate stent healing. The REMEDEE OCT study is currently randomizing 60 patients with an acute coronary syndrome to receive either a CD34 + antibody covered, sirolimus-eluting stent (Genous Combo) or an everolimus-eluting stent (Xience V or Promus) and evaluates the early stent healing response by OCT/OFDI at 2 month follow-up (ClinicalTrials.gov Identifier: NCT01405287).

## OCT Imaging After Stent Implantation: Tissue Characterization Around Stent Struts

While the resolution of OCT is substantially greater as compared to ultrasound and can be in the range of 10 μm axial and 20–40 μm lateral resolution, it does not allow detection of a single cell layer (such as the endothelium), that might become possible with the microOCT systems that have been examined in the experimental setting [29]. However, several studies have suggested a close relation between the detection of stent strut coverage by OCT as compared to histology [30•, 31, 32]. Moreover, there has been substantial interest in OCT image characteristics that may aid in the distinction between different tissues covering stent struts, i.e. fibrin coverage with thrombotic material vs neointimal coverage. Backscattering intensity and signal attenuation may provide information for the further discrimination of stent strut coverage tissue type, in particular a lower OCT signal intensity has been observed for fibrin-covered stent struts as compared to neointimal covered stent struts [30•, 31]. In a study of porcine coronary stent implantation we have observed that fibrin-rich tissue as detected early after porcine coronary stent implantation and confirmed by electron microscopy had a substantially lower OCT signal-intensity when compared to neointima-covered stent struts later after implantation, that were covered by smooth muscle cell-rich tissue with extracellular matrix containing collagens [30•]. Nakano et al. have recently reported in a study using stents obtained at autopsy that a lower signal intensity and a greater signal attenuation was observed for fibrin as compared to neointima-covered stent struts when examined by OFDI [31]. Furthermore, the luminal surface appears more irregular when stent struts are covered by fibrin accumulation as compared to neointima [30•, 31]. It is likely that the further characterization of the type of tissue coverage of the stent struts will be important, since only neointima, but not fibrin-coverage of stent struts can be considered as an effective stent healing. For example, a recent study in patients with STEMI has reported an OCT follow-up after 3–7 days and reported a high percentage of stent struts that were covered with a thin rim, making it likely that this may rather represent fibrin than neointimal stent strut coverage [33].

## OCT: A Valuable Tool for Evaluation of Vascular Response of Bioabsorbable Stents

The reproducibility of gray-scale IVUS was reported to be lower as compared with OCT for detection of qualitative findings after bioresorbable vascular scaffold (BVS) implantation [34]. OCT is capable of an accurate assessment of the polymeric struts, the changes in luminal and scaffold dimensions, and the quantification of neointimal hyperplasia [35••]. The amount of backscattering in OCT imaging depends on the material and the progress of strut biodegradation. Recently published studies have provided data with respect to safety and feasibility of bioresorbable stents as revealed by OCT [35••, 36]. Two years after bioabsorbable everolimus-eluting coronary stent implantation in the ABSORB study OCT imaging suggested that the stents had been incorporated into the vessel wall and was largely bioabsorbed; 34 % of the stent struts were no longer discernible at all by OCT after 2 years, suggesting that they had been completely bioabsorbed [35••]. The OCT/OFDI quantification of the healing process after bioabsorbable stent implantation has important differences as compared to the metal-scaffold stents. Strut core areas do not produce dorsal shadows which allows for a complete imaging of strut thickness [37, 38].

## OCT Examination of Stent Healing and Risk of Stent Thrombosis

Late and very late stent thrombosis is a rare, but feared complication after coronary stent implantation. Several causes can lead to late or very late stent thrombosis, including impaired stent healing (uncovered/malapposed stent struts), but also neoatherosclerosis and rupture within the stent [8•, 39]. As described above, autopsy studies have suggested that the percentage of uncovered stent struts is particularly high in patients who died from a stent thrombosis [8•, 39]. The clinical implications, however, of a certain percentage of uncovered or malapposed stent struts need to be further examined and evaluated. In this respect, a recent case-controlled study has suggested a substantially increased frequency and length of uncovered and malapposed stent struts as assessed by OCT in patients with late stent thrombosis after DES implantation (percentage of uncovered stent struts 12.2 % vs. 4.1 %*)* [40•]. In pathological studies the presence of >30 % uncovered stent-struts was highly predictive of in-stent thrombosis after DES implantation [8•]. Ozaki et al. reported, that incomplete stent apposition without neointimal hyperplasia was significantly associated with the presence of OCT-detected thrombus at follow-up, and may constitute a potent substrate for LIST [14].

Besides impaired stent healing, neoatherosclerosis may also contribute to clinical events late after DES implantation due to neointimal rupture as suggested by a recent study using OCT [41].

## OCT and Detection of Subclinical Dissections After Stent Implantation (Edge Dissections)

**Fig. 4:**
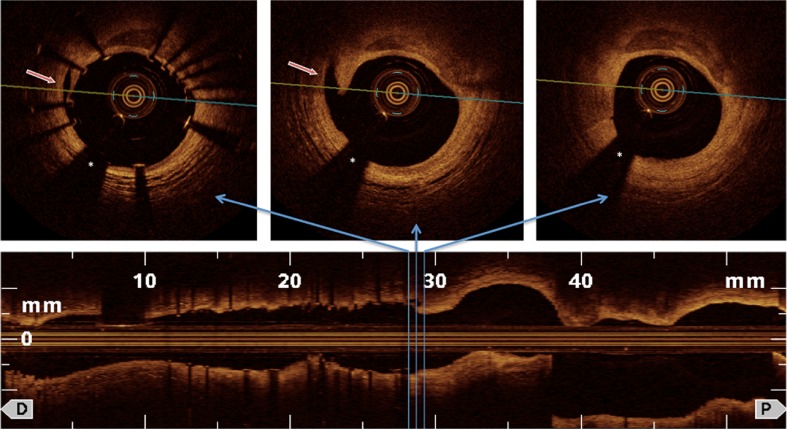
In stent dissection and edge dissection after coronary stent implantation that can be detected by OCT and frequently would not be seen by angiography or IVUS (*red arrows*). *Asterisk* indicates OCT catheter

Because of the high resolution of OCT small intimal dissections, also termed small intimal disruptions, are more frequently observed after coronary stent implantation by OCT as compared to what is detected by angiography or IVUS [42] (see example Fig. 4). Such subclinical dissections are detected within the stent, but often also at the stent edges (i.e. edge dissections) [43]. There is no evidence that such subclinical dissections as revealed by OCT should be “sealed” by additional overlapping stents [43]. On the contrary, multiple overlapping stents are known to be associated with stent strut malapposition and have been reported as a predictor of LIST after DES [44]. In a study analyzing the acute effects of coronary stenting by OCT in 80 vessels (and 73 patients), a stent edge dissection was detected in 20 vessels without clinical events during hospitalization [43]. A small study has reported the frequency of stent edge dissection as detected by OCT or IVUS during acute stent implantation and follow-up after approximately 6 months in 36 patients [45]. Notably, all OCT-detected stent edge dissections were healed without thrombus formation at the late follow-up [45], suggesting that small edge dissections should not prompt additional stent implantation.

A recent study using OCT, however, has suggested that intra-stent dissections (besides thin-cap fibroatheroma and intra-stent thrombus) were associated with a higher risk of periprocedural (type IVa) myocardial infarction in 50 patients with stable CAD or an NSTEMI [46].

## Future Perspectives/Conclusions

OCT is a feasible and safe imaging modality for the detailed evaluation of the acute and chronic vascular response after coronary stent implantation, allowing detection of stent strut coverage, strut malapposition, angiographically inapparent thrombus formation, and small intimal ruptures [32, 47–50]. OCT reveals much more detail after coronary stent implantation as compared to IVUS [51]. A remaining challenge is to better define the clinical implications of the observations made by OCT, that will likely require larger OCT studies and registries with long-term follow-up.
